# Broad-Spectrum, Potent, and Durable Ceria Nanoparticles Inactivate RNA Virus Infectivity by Targeting Virion Surfaces and Disrupting Virus–Receptor Interactions

**DOI:** 10.3390/molecules28135190

**Published:** 2023-07-04

**Authors:** Candace R. Fox, Kritika Kedarinath, Craig J. Neal, Jeremy Sheiber, Elayaraja Kolanthai, Udit Kumar, Christina Drake, Sudipta Seal, Griffith D. Parks

**Affiliations:** 1 Burnett School of Biomedical Sciences, College of Medicine, University of Central Florida, Orlando, FL 32827, USA; krkedari@utmb.edu (K.K.); jeremy.sheiber@ucf.edu (J.S.); griffith.parks@ucf.edu (G.D.P.); 2 Advanced Materials Processing and Analysis Center, Department of Materials Science and Engineering, University of Central Florida, Orlando, FL 32816, USA; craig.neal@ucf.edu (C.J.N.); elayaraja.kolanthai@ucf.edu (E.K.); ud486414@ucf.edu (U.K.); sudipta.seal@ucf.edu (S.S.); 3 Kismet Technologies, Orlando, FL 32792, USA; cdrake@kismettechnologies.com; 4 Nano Science Technology Center, University of Central Florida, Orlando, FL 32816, USA; 5 Biionix Cluster, College of Medicine, University of Central Florida, Orlando, FL 32816, USA

**Keywords:** anti-viral, virucidal, nanoparticles, disinfectant, rhinovirus, coronavirus, norovirus, parainfluenza

## Abstract

There is intense interest in developing long-lasting, potent, and broad-spectrum antiviral disinfectants. Ceria nanoparticles (CNPs) can undergo surface redox reactions (Ce^3+^ ↔ Ce^4+^) to generate ROS without requiring an external driving force. Here, we tested the mechanism behind our prior finding of potent inactivation of enveloped and non-enveloped RNA viruses by silver-modified CNPs, AgCNP1 and AgCNP2. Treatment of human respiratory viruses, coronavirus OC43 and parainfluenza virus type 5 (PIV5) with AgCNP1 and 2, respectively, prevented virus interactions with host cell receptors and resulted in virion aggregation. Rhinovirus 14 (RV14) mutants were selected to be resistant to inactivation by AgCNP2. Sequence analysis of the resistant virus genomes predicted two amino acid changes in surface-located residues D91V and F177L within capsid protein VP1. Consistent with the regenerative properties of CNPs, surface-applied AgCNP1 and 2 inactivated a wide range of structurally diverse viruses, including enveloped (OC43, SARS-CoV-2, and PIV5) and non-enveloped RNA viruses (RV14 and feline calicivirus; FCV). Remarkably, a single application of AgCNP1 and 2 potently inactivated up to four sequential rounds of virus challenge. Our results show broad-spectrum and long-lasting anti-viral activity of AgCNP nanoparticles, due to targeting of viral surface proteins to disrupt interactions with cellular receptors.

## 1. Introduction

Viruses represent major public health concerns, with ubiquitous (e.g., norovirus), seasonal (e.g., rhinovirus), and pandemic (e.g., SARS-CoV-2) viral pathogens imposing a huge burden on the healthcare industry and economy. Non-influenza-related infections, such as rhinovirus and coronavirus, account for ~$40 billion annually in US medical costs, with costs similar to that seen with chronic conditions like congestive heart failure and hypertension [1]. Viruses can be difficult to inactivate due to their inherent stability, small size, high ratio of infectivity per particle, and resistance to disinfectants [2,3]. Many respiratory virus particles can remain infectious on surfaces for long periods of time, including influenza virus [4], rhinovirus [5], and coronavirus [6]. Prior work has shown that SARS-CoV-2 remains infectious at room temperature for at least 5 days in a dried form and 14 days in solution [7], and can last for up to 7 days on a surgical mask [8]. For some viruses such as rhinovirus, a major route of transmission is via contaminated surface exposure to hands followed by nose and eyes exposure, ultimately leading to self-inoculation [9].

The stability and surface transmission of viruses is particularly problematic for very stable and highly infectious virus particles, such as those found with rhinovirus and norovirus. Rhinovirus is effectively transmitted by hands from external surfaces to mucosal surfaces to initiate an infection [9,10]. Norovirus is an enteric virus which is estimated to affect 1 in every 15 US residents each year as a leading cause of food-related illnesses [11] that can be transmitted by food handlers and contact with virus-contaminated surfaces, such as equipment and facilities in hospitals, nursing homes, and schools [12,13]. Thus, there is intense interest in the development of long-lasting, potent, and broad-spectrum antiviral disinfectants for high-touch surfaces.

Promising disinfectants are under development that are based on nanotechnology reviewed by [14], with attention directed to nanoparticles composed of a wide range of different materials. Broadly speaking, formulations of nanoparticles made from a variety of materials show anti-viral activities through either: (1) adsorption to virus surface structures (glycoprotein sites, lipid membrane), or by (2) oxidative damage due to generation of particle–surface-catalyzed reactive oxygen species (ROS) [15]. In the first case, it is proposed that nanoparticle binding to virions can produce a substantial force at the particle surface due to near-field effects and secondary forces [16], and this is sufficient to denature proteins or disrupt viral membranes [16,17]. Similarly, it has been demonstrated that nanoparticle formulations that include hydrophobic regions in a negatively charged organic macro-molecule can produce virucidal action on some enveloped viruses [18,19].

In contrast to the above adsorption-mediated inactivation, an alternative mechanism for other antimicrobial nanoparticle formulations is through the generation of ROS. In most instances, the nanoparticle-derived ROS is generated through the dissolution of a metal nanoparticle (e.g., silver or copper/copper oxide) via interaction with water or an organic substrate such as a lipid [20]. Alternatively, the ROS may be generated through a photocatalytic mechanism such as that of TiO_2_ [21], with a limitation being that ultraviolet light is required to mediate excitation.

Nanoparticles can be further modified to contain Ag or other metal ions (e.g., gold, zinc oxide, copper oxide) to increase anti-microbial activity, with some Ag-containing formulations having a high affinity for virions that is dependent on the size of the nanoparticle [22]. Ag nanoparticles can release Ag ions that can interact with viral proteins and form ROS [23]. Metal-modified nanoparticles have been proposed to reduce virus infectivity through a number of possible mechanisms, including inhibition of viral attachment, direct virus inactivation, oxidation of viral proteins, and degradation of the viral genome [reviewed by 14]. However, there are few examples of identification of the exact step in the virus growth cycle which is disrupted by Ag nanoparticles.

Cerium oxide (CeO_2_; ceria) nanomaterials are particularly attractive for use in antimicrobial applications [16,24,25]. A unique property of Ceria nanoparticles (CNPs) lies in their ability to undergo a redox reaction (Ce^3+^ ↔ Ce^4+^) at their surface. Importantly, Cerium oxide nanomaterials have the interesting property of being able to both scavenge and produce ROS [26,27]. When combined with Ag, these AgCNP formulations show an advantage over other nanomaterial compositions described in the literature. Advantages could be attributed to ceria’s surface redox states, and their ability to generate ROS without appreciable chemical dissolution and without requiring an external driving force, such as the ultra-violet excitation source necessary for TiO_2_ activity [21,28]. Due to the regenerative properties of these AgCNPs, the lifetime of the active material is extended, and the material’s disinfecting activities are effectively independent of environmental conditions or the availability of an added (finite) chemical substrate.

We previously synthesized two formulations of AgCNP nanoparticles—AgCNP1 and AgCNP2—by chemical methods chosen to maximize differences in particle character between the formulations as detailed in our previous publication [28]. Importantly, the materials differed in the relative amount of reduced state Ce^3+^ to oxidized Ce^4+^ in the sites at the material surface (AgCNP2 has ~two-fold greater Ce^3+^ population), and in the relative sizes and particle density of silver phases at a single ceria particle (AgCNP1: multiple small AgNPs across the CNP surface; AgCNP2: silver and ceria phases of comparable size in ~1:1 particle ratio) [22,27]. These properties are known to strongly influence the functional behaviors of both silver and ceria with respect to antimicrobial and ROS scavenging/production.

Our previous work established that AgCNP1 and AgCNP2 were selective in their ability to inactivate two prototypic enveloped and non-enveloped respiratory viruses in solution: AgCNP1 was potent against seasonal coronavirus OC43 (enveloped −ssRNA virus), while AgCNP2 was potent against rhinovirus 14 (RV14, non-enveloped +ssRNA virus) [28]. While AgCNP nanoparticles had potent antiviral activity [28], their antiviral mechanism of action could *a priori* be targeted at any of the different possible steps in the replication cycle, including attachment, penetration, uncoating, and gene expression.

Here, we have used biochemical and genetic approaches to test the hypothesis that the mechanism of AgCNP anti-viral activity was through the disruption of an early step in virus replication prior to intracellular viral gene expression. We show that AgCNP1 and AgCNP2 prevented coronavirus OC43 and parainfluenza virus type 5 (PIV5), respectively, from binding to their host cell receptors and induced virion aggregation. Sequence analysis of RV14 mutants which had gained resistance to AgCNP inactivation revealed substitutions in external regions of the viral capsid, consistent with a hypothesis that AgCNP disrupted the virion surface leading to defects in attachment to cells. Consistent with a mechanism of regeneration of activity, AgCNPs showed durable and long-lasting antiviral activity, with coated slides inactivating up to four sequential rounds of challenge with either the norovirus surrogate feline calicivirus (FCV) or RV14. Taken together, our work provides a mechanism for the anti-viral potency of AgCNP and highlights powerful and durable applications of broad-spectrum nanoceria-based surface disinfectants.

## 2. Results

### 2.1. Silver-Modified Nanoceria Inactivates Distinct Enveloped RNA Viruses through the Disruption of Virus–Receptor Interactions and Virus Aggregation

**Figure 1:**
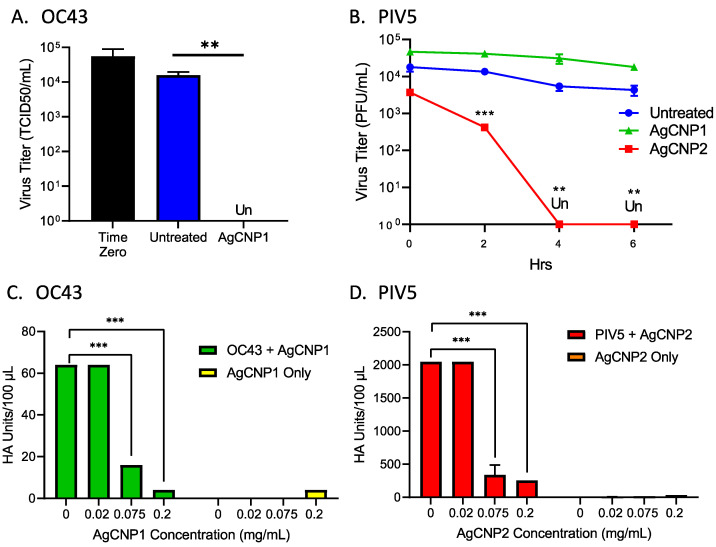
Silver-modified nanoceria inactivates enveloped RNA viruses OC43 and PIV5 and disrupts receptor binding. (**A**) Reactions of OC43 were prepared with buffer or with 0.2 mg/mL of AgCNP1. After a 4 h incubation, remaining infectivity was quantified by TCID_50_ assay. A sample taken immediately upon the addition of nanoparticles is indicated as time zero. (**B**) PIV5 was incubated with buffer (blue line), 0.2 mg/mL AgCNP1 (green line), or 0.2 mg/mL AgCNP2 (red line) for 0, 2, 4, and 6 h. Remaining infectivity was determined by plaque assays. (**C**) OC43 was combined with 0, 0.02, 0.075, and 0.2 mg/mL AgCNP1, followed by assaying for hemagglutination activity with red blood cells to determine virus–receptor binding. The same concentrations of AgCNP1 incubated with red blood cells alone were included as control reactions (yellow bars). (**D**) Reactions of PIV5 were prepared with 0, 0.02, 0.075, and 0.2 mg/mL AgCNP2, and hemagglutination assays were performed. For all graphs, values are the mean of three independent samples, with error bars representing the standard deviation. Un denotes undetectable infectivity and **, and *** indicate *p*-values of <0.01, and <0.001, respectively, comparing untreated to indicated treatment.

Two formulations of AgCNP nanoparticles—AgCNP1 and AgCNP2—were synthesized as described in materials and methods and tested in mechanistic studies for the inactivation of viruses in aqueous solutions. An example of this inactivation of the enveloped, positive-sense RNA virus coronavirus OC43 by AgCNP1 is shown in Figure 1A [28]. Here, ~10^5^ of 50% Tissue Culture Infectious Dose (TCID_50_) units of OC43 were either left untreated or treated with 0.2 mg/mL AgCNP1 for 4 h (hrs), and the remaining infectious OC43 was then quantified by TCID_50_ assays. AgCNP1 effectively inactivated OC43 infectivity to below the detectable limits.

We extended these analyses to a structurally different enveloped virus—the negative-sense RNA virus parainfluenza virus type 5 (PIV5). Liquid reactions were prepared to include ~10^4^ Plaque Forming Units (PFU) of PIV5 with 0.2 mg/mL of either AgCNP1 or AgCNP2 or a buffer alone control. At time zero, 2, 4, and 6 h after mixing, the remaining virus infectivity was determined by plaque assay. As shown in Figure 1B, a 4 h incubation of PIV5 with AgCNP2 resulted in complete virus inactivation, whereas AgCNP1 treatment appeared to have the same effect on infectious PIV5 as buffer alone. Taken together, these data demonstrate that OC43 and PIV5 are sensitive to inactivation in liquid by AgCNP1 and AgCNP2, respectively.

Some enveloped viruses contain a spike protein which can bind to sialic acid on the surface of cells. As such, hemagglutination assays (HA) are a well-established method to quantify virus particle attachment to cells [29], through assays of virion-mediated cross-linking of red blood cells (RBCs) to cause agglutination. The coronavirus OC43 spike glycoprotein binds to sialic acid receptors and can be quantified by HA assays [30,31]. To determine if the binding of the OC43 virus to red blood cells was disrupted by treatment with nanoparticles, OC43 in solution was either left untreated or treated with increasing concentrations of AgCNP1, and the remaining receptor binding was tested in HA assays on RBCs. As shown in Figure 1C, control samples of AgCNP1 with RBCs alone did not induce agglutination (right side, yellow bars). Untreated OC43 had an HA titer of ~64 HA units, whereas reactions with 0.075 and 0.2 mg/mL AgCNP1 reduced the OC43 HA titer to ~10 and 2 units, respectively. These results support the contention that AgCNP1 treatment disrupts the interactions of OC43 binding to a host cell receptor.

The PIV5 glycoprotein hemagglutinin-neuraminidase (HN) glycoprotein is responsible for binding virions to sialic acid receptors on host cells [32]. To test for AgCNP-mediated disruption of PIV5 receptor binding, reactions were prepared with PIV5 and buffer only, AgCNP2 only, and PIV5 treated with 0.02, 0.075, or 0.2 mg/mL AgCNP2. After incubation, the remaining HA was then tested on RBCs. As shown in Figure 1D, RBC treatment with AgCNP2 alone did not show HA activity (right side, orange bars). By contrast, treatment of PIV5 with 0.075 and 0.02 mg/mL AgCNP2 reduced PIV5 HA titers from ~2000 in the untreated sample to 250 and 200, respectively. These results indicated that AgCNP2 prevented PIV5 from binding to RBC indicator cells in a dose-dependent manner. Taken together, these data indicate that both AgCNP1 and 2 treatment of OC43 and PIV5, respectively, disrupted virus–receptor interactions.

### 2.2. Silver-Modified Nanoceria Induces Aggregation of Distinct Enveloped RNA Viruses

**Figure 2:**
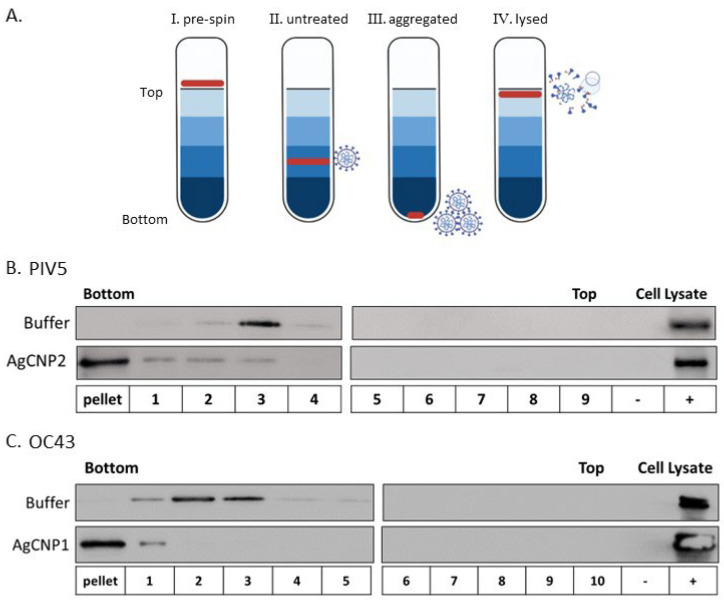
Silver-modified nanoceria induces PIV5 and OC43 virion aggregation. (**A**) Possible outcomes of the sucrose gradient assay diagram. Virus layered on the top of the gradient (panel I) will migrate to a certain position in the sucrose gradient (example panel II), depending on virus type. Virion aggregates pellet to the bottom of the gradient (example panel III), whereas lysed or disrupted virions remain near the top of the gradient (example panel IV). (**B**) PIV5 virions were treated with buffer alone (**top** panel) or treated with 0.4 mg/mL AgCNP2 (**bottom** panel) for 4 h before loading onto pre-equilibrated sucrose gradients. After centrifugation, fractions were collected from the bottom of the tube and the pellet was resuspended. Samples were analyzed by western blotting for PIV5 viral protein P. (**C**) OC43 was treated with buffer alone (**top** panel) or treated with 0.6 mg/mL AgCNP1 (**bottom** panel). After 4 h incubation, reactions were centrifuged on sucrose gradients as described above for panel B, with fractions being analyzed by western blotting for OC43 viral protein NP. For both B and C, negative (−) and positive (+) cell lysate controls for western blotting were also analyzed. Respective cell lysate controls included H1299 cells either mock infected or infected with PIV5 (**B**) or H1299 cells either mock infected or infected with OC43 (**C**).

The above data showing nanoparticle-mediated inhibition of virus–receptor interactions could be explained by changes in the integrity of the virion particles (e.g., virion lysis), or alternatively, by the formation of aggregates of virion particles. Sucrose gradient centrifugation was used to distinguish between these hypotheses [33,34]. As shown schematically in Figure 2A, intact virions migrate to a characteristic position in sucrose gradients depending on the particular virus (outcome II, untreated). It is expected that virion aggregates will pellet to the bottom of the gradient (outcome III), whereas lysed virions will float to the top of the gradient (outcome IV; [33]). To test virion integrity, reactions were prepared to include PIV5 only or AgCNP2-treated PIV5. After 4 h incubation, reactions were centrifuged on pre-equilibrated sucrose gradients, and gradient fractions and pellets were analyzed by western blotting for the position of the virion-associated PIV5 P protein. As shown in Figure 2B, untreated PIV5 virions were found to sediment largely to gradient fraction 3, whereas AgCNP2-treated PIV5 virions were found largely as an aggregate which had pelleted to the bottom of the tube. Similarly, buffer-treated OC43 was found largely in gradient fractions two and three as detected by the presence of viral protein NP (Figure 2C). By contrast, AgCNP1-treated OC43 was found as an aggregate in the pellet of the sucrose gradient. These data support the hypothesis that AgCNP1 and AgCNP2 treatment of enveloped OC43 and PIV5 viruses, respectively, resulted in the disruption of virus–receptor binding through induction of virion aggregation.

### 2.3. Generation of RV14 That Is Resistant to AgCNP2 Inactivation and Identification of Resulting Genomic Changes

**Figure 3:**
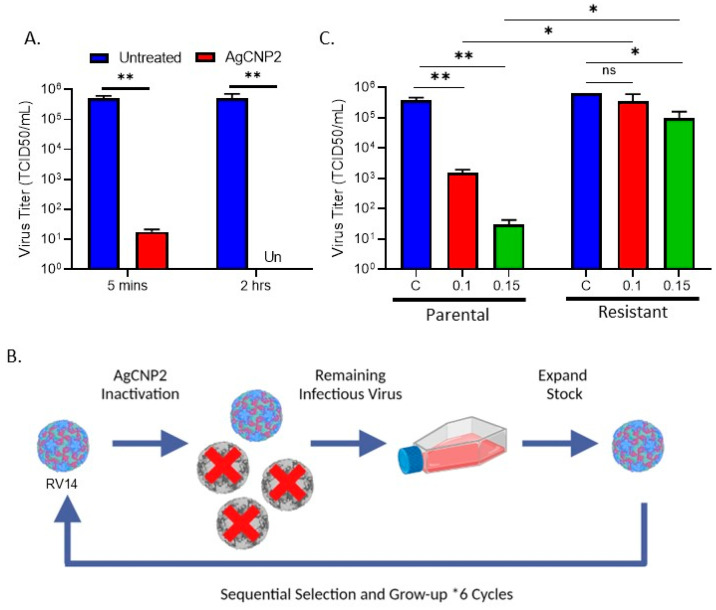
Selection for RV14 that has gained resistance to AgCNP2 inactivation. (**A**) Liquid solution reactions of RV14 were incubated at room temperature for 5 min or 2 h with buffer alone or with 0.3 mg/mL AgCNP2. Remaining infectivity was determined by TCID_50_ assay. (**B**) Schematic diagram of approach to generate AgCNP2-resistant RV14 by sequential rounds of partial inactivation with AgCNP2 as detailed in materials and methods, * indicates times 6 selection cycles. (**C**) Parental and AgCNP2-selected RV14 were incubated for 2 h with buffer alone, 0.1 mg/mL AgCNP2, or with 0.15 mg/mL AgCNP2. Infectivity was determined by TCID_50_ assay. For all graphs, values are the mean of three independent samples, with error bars representing the standard deviation. Un denotes undetectable and ns indicates no significance. * and ** indicate *p*-values of <0.05 and <0.01, respectively.

Our prior work showed that the non-enveloped positive-sense RNA virus rhinovirus (RV14) was very sensitive to inactivation by AgCNP2. An example of this anti-viral activity in liquid reactions is shown in Figure 3A. Here, ~5 × 10^5^ TCID_50_ units of RV14 were left untreated or treated with 0.3 mg/mL AgCNP2. After 5 min and 2 h, samples were quantified for infectivity by TCID_50_ assays. As shown in Figure 3A, as little as 5 min of incubation with AgCNP2 was sufficient to inactivate ~4.5 log TCID_50_ units of RV14 and a 2 h treatment inactivated RV14 to undetectable (Un) limits.

Due to the lack of available assays for RV14 receptor binding, we used an unbiased approach to determine the mechanism of inactivation by AgCNP2. We tested the hypotheses that serial passage of AgCNP2-treated RV14 would result in virus that was resistant to inactivation by AgCNP2, and that this would correlate with amino acid (AA) changes in the viral capsid region. As shown in the Figure 3B schematic, parental RV14 was processed through six sequential rounds of suboptimal AgCNP2 treatment, each of which was followed by expansion of the remaining infectious virus as described in the materials and methods. Complete AgCNP2 resistance was observed after three selection rounds, with partial resistance seen following the second selection round. The susceptibility of the parental RV14 versus the selected RV14 to AgCNP2 inactivation was tested. Here, ~5 × 10^5^ TCID_50_ units of parental or selected RV14 were treated for 2 h with buffer alone (C) or with 0.1 or 0.15 mg/mL AgCNP2, and the remaining infectivity was determined. As shown in Figure 3C, parental RV14 showed a dose-dependent inactivation of infectivity, with 0.15 mg/mL AgCNP2 treatment reducing infectivity by 10^4^ TCID_50_ units (green bar). In striking contrast, the same nanoparticle treatment of the selected RV14 viruses showed a reduction of only 0.5 log of infectivity (green bars, Figure 3C); the comparative infectivity remaining after AgCNP2 treatment between parental and selected RV14 was ~10^4^ TCID_50_ units. These data indicate that sequential rounds of treatment of RV14 with AgCNP2 leads to the enrichment of a virus population that is very resistant to inactivation by these nanoparticles.

**Figure 4:**
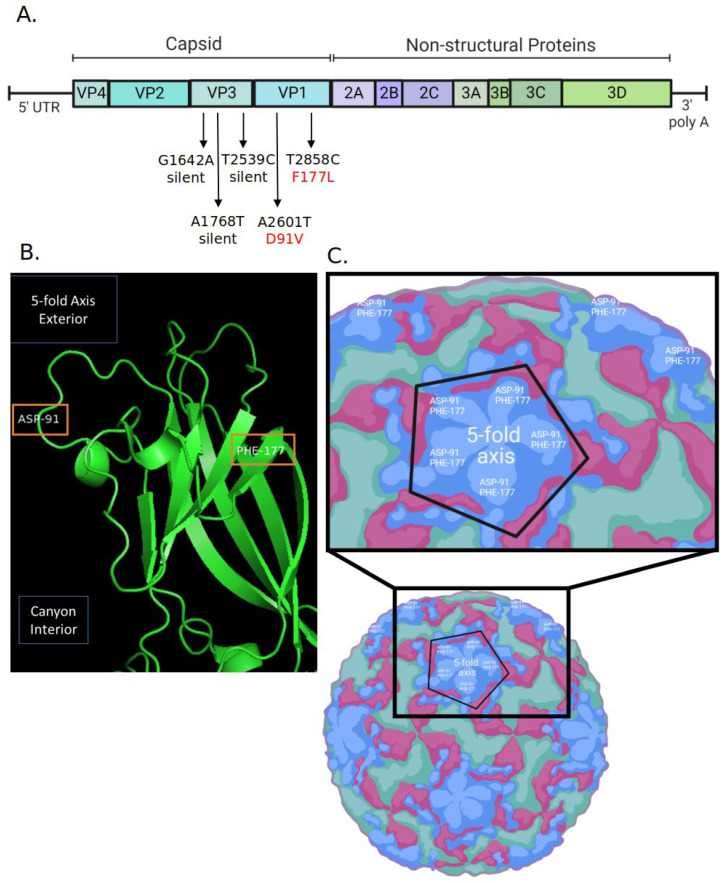
Predicted amino acid changes between parental and AgCNP2-resistant RV14 RNA map to the external surface of the RV14 capsid. (**A**) Schematic diagram of the RV14 genome, showing the location of nucleotide sequence differences between parental and AgCNP2-resistant RV14. Three of the five RNA sequence differences were found in the *VP3* gene, but did not change the predicted coding of the AA. By contrast, 2 out of the 5 RNA sequence changes were in the *VP1* gene and resulted in AA alterations F177L and D91V, as denoted in red. UTR: untranslated region. (**B**) The predicted tertiary amino acid ribbon structure of parental RV14 VP1 is shown with the location of the two amino acid changes between parental and AgCNP2-resistant RV14, shown with orange boxes. (**C**) A schematic of the RV14 virion showing the predicted location of the 5-fold axis with 5 copies of the aspartic acid 91 (ASP-91) and phenylalanine 177 (PHE-177) changes on the exterior surface of VP1.

The RNA genomes of parental and AgCNP2-resistant RV14 were sequenced, with reads aligned to the archived RV14 reference sequence [35]. Figure 4A shows a schematic diagram of the RV14 genome with a 5′ end capsid region comprised of four viral capsid proteins VP1, VP2, VP3, and VP4 [36], and a 3′ region composed of non-structural proteins. Figure 4A also shows the location of nucleotide changes between parental and AgCNP2-resistant genomes detected by sequence analysis and their predicted amino acid substitutions. Remarkably, within the ~7000 nucleotide-long genomes, there were only five nucleotide sequence differences found between the parental and AgCNP2-resistant viruses. Three of the five mutations were in the coding region of *VP3*, but these were silent alterations that did not change the predicted amino acid sequence. Most importantly, two out of the five differences between nucleotide sequences of the RV14 genomes were in the *VP1* coding region, resulting in two predicted amino acid alterations as shown in red in Figure 4A: Aspartic acid 91 to Valine (D91V) and Phenylalanine 177 to Leucine (F177L).

The location on the virion particle of the two predicted amino acid changes between parental and AgCNP2-resistant viruses was mapped through protein modeling using Phyre2 [37] and visualized on the PyMOL software [38]. Figure 4B shows the tertiary amino acid ribbon structure of parental RV14 VP1 protein and the location of two amino acids that are altered in the AgCNP2-resistant RV14, shown as orange box highlights. These amino acid changes are predicted to be on the surface of the virion, located away from the central interior core and the canyon which is the site of key residues involved in receptor interactions [39]. As shown in the schematic in Figure 4C, the icosahedral symmetry of the RV14 capsid places multiple copies of the VP1 subunit at the twelve five-fold axes of the virion—thus, the two amino acid changes are predicted to be repeated across the virion spheroidal structure. Taken together, the sequence analysis of the AgCNP-resistant virus that predicts altered amino acids on the external surface of RV14 virions is consistent with a mechanism of AgCNP inactivation that targets the inactivation of the capsid structure and disruption of an early step in the virus replication cycle. It is noteworthy that one of these predicted AA substitutions (D91V) is also associated with capsid changes that allow RV14 to escape neutralization by monoclonal antibodies [40].

### 2.4. Surface-Dried Silver-Modified Nanoceria Inactivated Structurally Distinct Enveloped RNA Viruses

**Figure 5:**
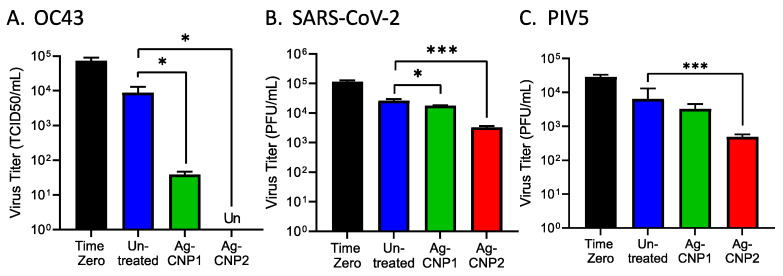
Surface-dried silver-modified nanoceria inactivates enveloped RNA viruses OC43, SARS-CoV-2, and PIV5. (**A**–**C**) Glass slides were left uncoated (blue bars) or coated with 0.1 mg of either AgCNP1 (green bars) or AgCNP2 (red bars) and then dried. Slides were inoculated with the indicated doses of the individual viruses OC43 (**A**), SARS-CoV-2 (**B**), and PIV5 (**C**), and virus was either recovered immediately after inoculation (time zero, black bars) or incubated at room temperature for two hrs. Remaining infectious virus was recovered from the slides and quantified by either TCID_50_ assay (OC43) or plaque assay (SARS-CoV-2, PIV5). Values are the mean of three independent samples, with error bars representing the standard deviation. * and *** indicate *p*-values of <0.05 and <0.001, respectively, comparing untreated to indicated treatment.

We extended the above mechanistic studies in liquid to address the practical question of whether AgCNP anti-viral activity was retained on hard surfaces, which can be a major source of viral transmission. To determine the extent of antiviral activity of surface-dried silver nanoceria particles, glass slides were treated with 0.1 mg of either AgCNP1 or AgCNP2, dried, and then challenged with ~8 × 10^4^ TCID_50_ units of the enveloped coronavirus OC43. Slides were immediately washed with 0.5 mL cell culture media as a time-zero sample (Figure 5A, black bar). After a 2 h incubation, slides were similarly processed for remaining infectivity. As shown in Figure 5A, untreated control slides retained 1 × 10^4^ TCID_50_ infectious units of OC43 (blue bar), whereas AgCNP1-coated slides reduced infectious OC43 to only ~50 TCID_50_ units/mL (green bar). Most strikingly, AgCNP2-coated slides resulted in levels of infectious OC43 that were below detection.

Using a similar approach, we determined if the enveloped RNA viruses SARS-CoV-2 and PIV5 were also susceptible to inactivation by AgCNP dried on surfaces. AgCNP1- or 2-coated slides were challenged with ~1 × 10^5^ PFU of SARS-CoV-2 and then processed immediately or after a 2 h incubation. Untreated and AgCNP1-coated slides showed a reduction in infectious SARS-CoV-2 to ~1–2 × 10^4^ PFU (Figure 5B), whereas AgCNP2-coated slides reduced virus infectivity to ~5 × 10^3^ PFU. Similar results were seen with PIV5, where AgCNP2-coated slides reduced PIV5 titers from ~2 × 10^4^ PFU to ~5 × 10^2^ PFU (Figure 5C). Thus, antiviral potency against these three enveloped RNA viruses is retained when AgCNP is dried on hard surfaces.

### 2.5. A Single Coating of Silver-Modified Nanoceria Inactivates Multiple Rounds of RV14 Challenge

**Figure 6:**
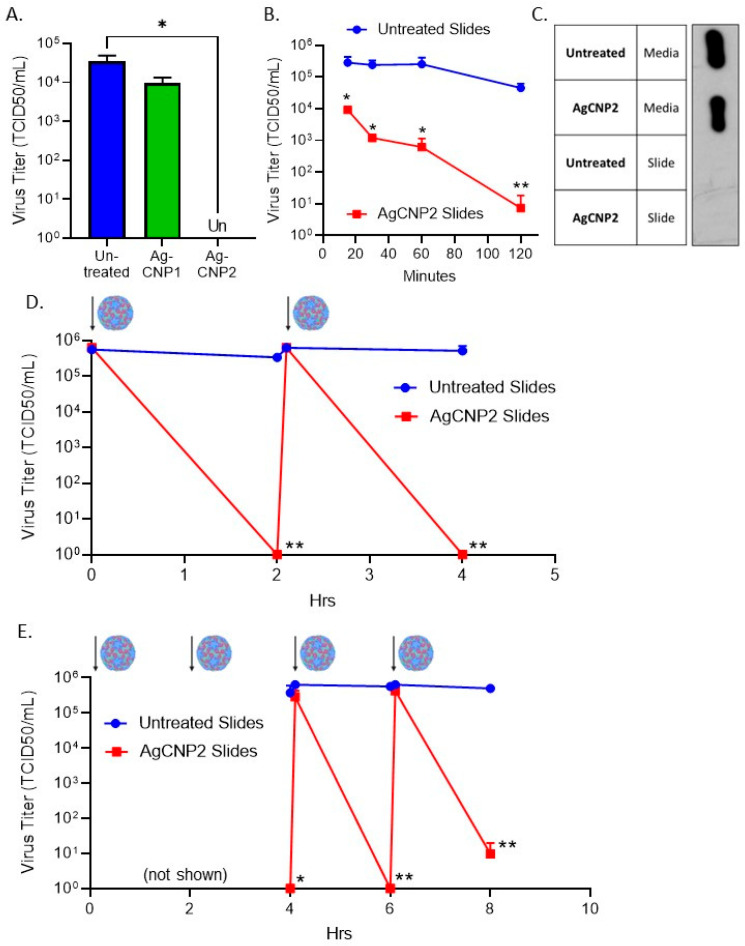
A single coating of AgCNP2 can inactivate multiple rounds of RV14 re-challenge. (**A**) Slides were left uncoated or coated with 0.1 mg of either AgCNP1 or AgCNP2 and then dried. Slides were inoculated with 5 × 10^4^ TCID_50_ units of RV14 and incubated at room temperature for two hrs. Remaining infectious virus was recovered from the slides and quantified by TCID_50_ assay. (**B**) Slides were left uncoated or coated with 0.0425 mg of AgCNP2 and dried. Slides were challenged with 5 × 10^5^ TCID_50_ units of RV14, and remaining infectivity on slides after 15, 30, 60, and 120 min was determined by TCID_50_ assays. (**C**) Media and slide surfaces collected after 2 h incubation were treated with protein lysis buffer and samples were analyzed by western blotting for RV14 viral protein VP3. (**D**) Sets of slides were left uncoated or treated with 0.1 mg of AgCNP2 and dried. Approximately 1 × 10^6^ TCID_50_ units of RV14 were applied to all slides as denoted by the cartoon viruses and arrows. One set of slides was processed immediately upon RV14 application (time zero). After 2 h incubation, a second set of slides was processed and associated RV14 infectivity was determined by TCID_50_ assay. The third set of unprocessed slides received a new re-application challenge with ~1 × 10^6^ TCID_50_ units of RV14. Slides processed immediately upon the second application of RV14 are indicated. Slides were then incubated for an additional 2 h, processed, and remaining RV14 infectivity on the slides was quantified and expressed as the 4 h time point. (**E**) Slides were treated and challenged with RV14 for 2 rounds as described in panel (**D**) (not shown, but indicated by arrows). At 4 h, slides were processed and remaining infectious RV14 was determined. Slides were further re-challenged a third and a fourth time with approximately 1 × 10^6^ TCID_50_ units of RV14, with incubation times after each challenge of an additional 2 h, followed by processing and quantifying remaining RV14 infectivity. For all figures, values are the mean of three independent samples, with error bars representing the standard deviation. * and ** indicate *p*-values of <0.05 and <0.01, respectively, comparing untreated to indicated treatment.

Many non-enveloped viruses are efficiently transmitted through contact with contaminated surfaces, highlighting the practical application of AgCNPs as anti-viral agents for the treatment of surfaces. The non-enveloped positive-sense RNA virus RV14 was tested for sensitivity to AgCNP inactivation on surfaces. As shown in Figure 6A, AgCNP1-coated slides did not significantly reduce RV14 infectivity from a starting input value of 5 × 10^4^ TCID_50_ units/mL. Importantly, however, AgCNP2-coated slides reduced this input RV14 infectivity to undetectable levels (Figure 6A). To define the kinetics of RV14 inactivation by dried AgCNP2, slides were either left uncoated or coated with AgCNP2. After drying, slides were challenged with ~5 × 10^5^ TCID_50_ units of RV14 and then processed at 15, 30, 60, or 120 min for quantifying the remaining infectious RV14. As shown in Figure 6B, AgCNP2-coated slides reduced RV14 titers by more than 1 log to 1 × 10^4^ TCID_50_ units by 15 min and continued to reduce titers to ~10 TCID_50_ units within 120 min.

One possible explanation for the reduced RV14 titer from AgCNP2-coated slides is that infectious virus remained bound to the slide and was not detaching during slide processing and washing. To determine where virus mass resided during and after slide processing, untreated and AgCNP2-coated slides were incubated with RV14 for 2 h. Media-washed slides and slide surfaces were treated with protein lysis SDS buffer to recover protein, and lysates were analyzed by western blotting for the presence of RV14 VP3. As shown in Figure 6C, RV14 capsid protein was found predominantly in the media wash from both the untreated and AgCNP2-coated slides, with no detectable RV14 protein retained on the slides. These data show that the mass of the virus is efficiently recovered from the slides during processing and loss of recovered infectivity is not due to RV14 particles remaining on the slides.

To test the ability of a single coating of nanoparticles to inactivate multiple challenges with infectious RV14, two sets of slides were left untreated or treated with AgCNP2. After drying, all slides were challenged with ~1 × 10^6^ TCID_50_ units of RV14. One set of slides was processed at time zero to determine the maximum recovered virus, which was slightly less than 1 × 10^6^ TCID_50_ units (Figure 6D). All other slides were incubated for 2 h, whereby the next set of slides was processed to show AgCNP2-coated slides completely reduced RV14 to below detectable levels. The remaining slides were then re-challenged with ~1 × 10^6^ TCID_50_ units of RV14. After another 2 h, slides were then processed as 4 h samples. As shown in Figure 6D, nearly the same amount of infectious RV14 was recovered from untreated slides, whereas AgCNP2-coated slides reduced RV14 to below detectable levels. These results show that one application of AgCNP2 was sufficient to inactivate two sequential rounds of RV14 challenge.

To determine if one application of AgCNP2 dried could inactivate four sequential rounds of RV14 challenge, untreated or AgCNP2-coated slides were challenged with RV14 as described above, except slides at time zero, 2 h, and immediately after RV14 re-challenging were not processed for infectivity. Subsequent samples were processed for remaining RV14 infectivity every 2 h. Consistent with the above results, AgCNP-coated slides completely reduce RV14 titers to below detectable limits after two RV14 challenges (4 h timepoint in Figure 6E). Slides were then challenged with RV14 for a third time and slides were immediately processed, followed by a 4th challenge with RV14. Remarkably, after four sequential rounds of virus challenge, AgCNP2-coated slides were still able to reduce RV14 titer from ~1 × 10^6^ TCID_50_ units to 10 TCID_50_ units within a 2 h period. Taken together, these data demonstrate the potent anti-viral activity of AgCNP2 dried on a surface, even after four rounds of infectious virus challenge.

### 2.6. A Single Coating of Silver-Modified Nanoceria Inactivates Multiple Rounds of FCV Challenge

**Figure 7:**
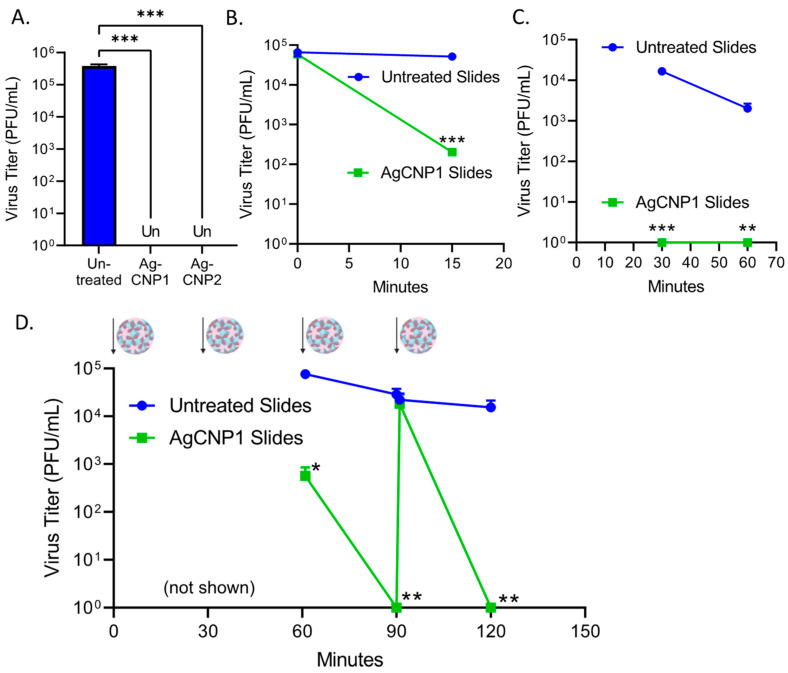
A single coating of AgCNP1 completely inactivates four sequential rounds of FCV challenge. (**A**) Slides were left uncoated or coated with 0.1 mg of either AgCNP1 or AgCNP2 and then dried. Slides were inoculated with 5 × 10^5^ of FCV and incubated at room temperature for two hr. Remaining infectious virus was recovered from the slides and quantified by plaque assay. (**B**,**C**) Slides were left uncoated or coated with 0.1 mg AgCNP1 and dried. Approximately 8 × 10^4^ PFU of FCV was applied to the slides and incubated for 0 and 15 min (**B**) or 30 and 60 min (**C**). After the indicated incubation time, slides were washed, vortexed in media, and remaining infectious FCV was quantified. (**D**) Three sets of slides were left uncoated or coated with 0.1 mg AgCNP1. After drying, ~1 × 10^5^ PFU of FCV was applied to the slides as denoted by the cartoon virus and arrow and incubated for 30 min. Slides were then challenged with a second round of FCV and incubated for an additional 30 min. One set of slides was processed, remaining infectious FCV was quantified by plaque assay, and data are expressed as the 60 min time point. The second set of unprocessed slides were challenged for a third time with FCV and slides were immediately processed and expressed as 60 min. Slides were incubated for an additional 30 min, followed by processing, and infectious FCV was determined and expressed as the 90 min time point. Slides were then re-challenged for a fourth time with FCV and slides were immediately processed and expressed as 120 min time point. Values are the mean of three independent samples, with error bars representing the standard deviation. *, **, and *** indicate *p*-values of <0.05, <0.01, and <0.001, respectively, comparing untreated to indicated treatment.

Feline calicivirus (FCV) is a positive-sense non-enveloped RNA virus that is recognized as a surrogate for human norovirus [41,42], with both viruses exhibiting difficulty in being inactivated on surfaces. By contrast to the differences in sensitivity of RV14 to AgCNP1 versus AgCNP2, FCV infectivity was very sensitive to both AgCNP1 and 2. This is shown in Figure 7A, where slides treated with either AgCNP1 or 2 reduced the input 5 × 10^5^ PFU of FCV to undetectable levels after a 2 h incubation. To define the kinetics of AgCNP1-mediated FCV inactivation on surfaces, slides were either left uncoated or coated with AgCNP1 and then challenged with ~1 × 10^5^ PFU of FCV. Slides were processed at 30- and 60-min incubation. As shown in Figure 7B,C, AgCNP1-coated slides reduced FCV titer by almost 3 logs to ~1 × 10^2^ PFU within 15 min of incubation (B), and completely reduced FCV titers below detectable limits after a 30 min incubation (C).

We tested the ability of one application of AgCNP1 coating of slides to inactivate multiple rounds of challenge with FCV virus, through the approach described above for RV14. As shown in Figure 7D, untreated slides or slides given a single coating of AgCNP1 were sequentially challenged with ~1 × 10^5^ PFU of FCV and incubated for 30 min before a subsequent re-challenge, as indicated by the virion and arrows. After each challenge, a set of slides was processed for recovery of input virus and at 2 h for the recovery of treated virus. Strikingly, Figure 7D shows that a single coating of slides with AgCNP1 reduced FCV titer to undetectable limits within ~30 min, even after four rounds of fresh virus challenge.

### 2.7. AgCNP2-Coated Slides Inactivate Both FCV and RV14 in a Mixed Virus Inoculum as Effectively as in Individual Virus Challenges

**Figure 8:**
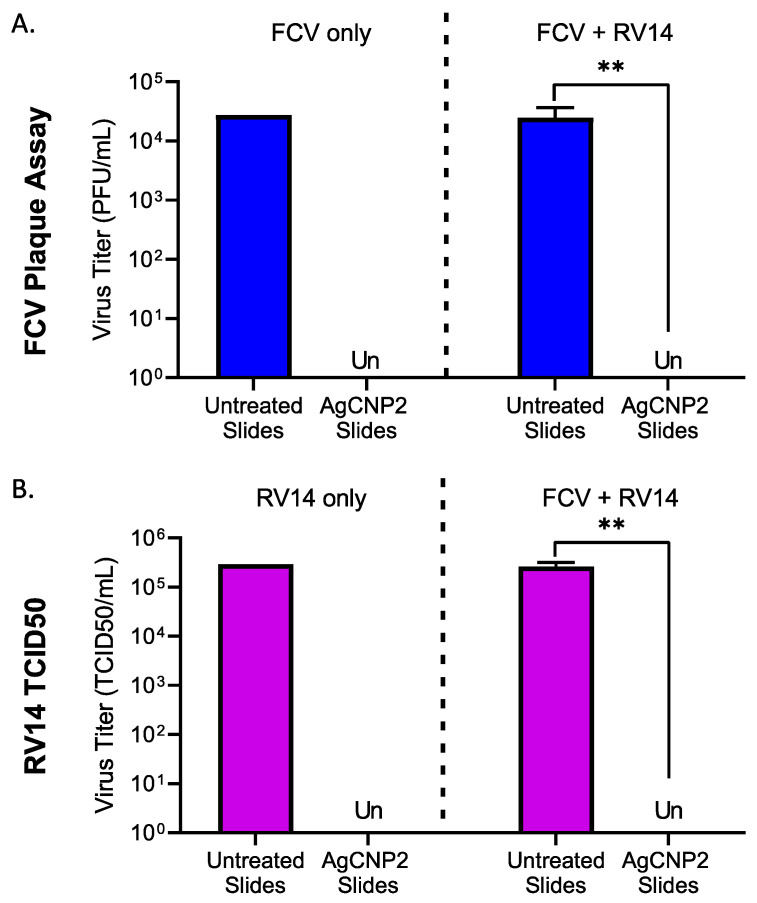
AgCNP2-coated slides inactivate both FCV and RV14 in a mixed virus inoculum as effectively as in individual virus challenges. (**A**,**B**) Three sets of slides were left uncoated or coated with 0.1 mg AgCNP2 and dried. One set of slides was challenged individually with ~5 × 10^4^ PFU of FCV, and a second set was challenged individually with ~1 × 10^5^ TCID_50_ units of RV14. A third slide set received a mixed inoculum of both FCV and RV14 at the above doses. After 2 h at room temperature, slides were vortexed in media and remaining infectious virus was collected. FCV titers were determined by plaque assay on CRFK which do not support RV14 infections. Remaining RV14 infectivity was determined by TCID_50_ assays on HeLa cells which do not support FCV infections. Individual FCV- and RV14-only virus controls were performed as single samples. Mixed inoculum values are the mean of three independent samples, with error bars representing the standard deviation. Un denotes undetectable and ** indicates *p*-value < 0.01 comparing untreated to indicated treatment.

We tested the hypothesis that a single coating of AgCNP2 could inactivate a mixture of RNA viruses without losing potency. This experiment took advantage of the cell-type susceptibility of feline cells to FCV but not to RV14 infection and, conversely, the susceptibility of human HeLa cells to RV14 but not to FCV infection. Slides were left untreated or coated with AgCNP2 and dried. Slides were challenged individually with ~3 × 10^4^ PFU of FCV, ~2 × 10^5^ TCID_50_ units of RV14, or a combination of the same amounts of the individual viruses together. Slides were incubated for 2 h and then processed for the remaining infectious virus using their respective indicator cells. Consistent with our previous data, AgCNP2-coated slides inactivated FCV alone (Figure 8A, left panel) and RV14 alone (Figure 8B, left panel). Remarkably, AgCNP2-coated slides completely inactivated both FCV (Figure 8A, right panel) and RV14 (Figure 8B, right panel) in the mixed virus challenge. These data demonstrated AgCNP2 was capable of inactivating two different viruses at the same time without a preference for the inactivation of one virus versus the other virus.

## 3. Discussion

There is intense interest in developing novel materials with broad-spectrum antimicrobial activities. Nanoparticle-based virucidal surface coatings are promising potent disinfectants to combat virus spread and disease [43]. Here we demonstrate that Ag-containing cerium nanoparticles (nanoceria) have potent antiviral activity against a range of diverse enveloped (OC43, SARS-CoV-2, PIV5) and non-enveloped (RV14, FCV) RNA viruses. The goals of this current study were to (1) test the hypothesis that the mechanism of AgCNP anti-viral activity was through disruption of virion surfaces resulting in defects in early stages of viral replication, e.g., attachment, and (2) demonstrate the durability, potency, and practicality of AgCNP nanoparticles when dried on hard surfaces. Taken together, our results support the proposal that AgCNP nanoparticles have broad-spectrum, durable, and long-lasting anti-viral activity against structurally diverse RNA viruses by targeting surface proteins of the virion, which can result in aggregation and defects in binding to cellular receptors.

AgCNP-mediated inactivation of RNA virus infectivity could *a priori* involve any number of possible mechanisms, including damaging genomic nucleic acid, changing virion-associated viral polymerase activity, disrupting virion integrity, or altering interactions with cellular receptors and other early phases of the viral replication cycle (e.g., penetration, uncoating). In the case of HIV, Ag nanoparticles can bind to the viral gp120 glycoprotein and prevent attachment to host receptor CD4 [44], and silver nanoparticles have been shown to prevent non-enveloped poliovirus-induced cytopathic effect (CPE) through mechanisms which remain to be elucidated [45]. Our biochemical mechanistic analyses clearly showed that nanoceria treatment of enveloped coronavirus OC43 and parainfluenza virus PIV5 resulted in defects in the binding of virions to the cellular receptor sialic acid. This indicates that inactivation is not through disruption of a post-attachment step (e.g., penetration, uncoating, gene expression), but rather a direct defect in the first step in virus growth—interactions with cellular receptors. In principle, this defect could be through disruption of virion integrity and disassembly (e.g., lysis), through small changes to the virion coat that alter receptor interactions, or through the assembly of virions into non-infectious aggregates which no longer properly access cellular receptors. Results from our gradient analysis of AgCNP-treated OC43 and PIV5 are consistent with the formation of large aggregates that sediment to the bottom of the centrifuge tubes, with no direct evidence for virion disruption by AgCNP. It is noteworthy that this mechanism by which AgCNP inactivates these two enveloped RNA viruses is similar to the formation of large aggregates that is seen during the inactivation of some viruses by complement innate immune pathways, as has been shown for the inactivation of enveloped viruses PIV5, vesicular stomatitis virus, and influenza virus [33,46,47].

Our most striking finding was the genetic selection of a stock of RV14 which had become ~10^4^-fold more resistant to inactivation by AgCNP compared to the parental RV14 stock. Sequence analyses indicated that the AgCNP-resistant virus had AA aspartic acid 91 (ASP-91) altered to valine (D91V) and phenylalanine 177 (PHE-177) altered to leucine (F177L). Rather than being buried within the capsid structure, these AA changes map to the exterior face of VP1 on the surface of the RV14 virion. Presumably, AgCNP (or its chemical byproducts) had direct access to these particular AAs in the parental RV14 capsid to cause inactivation of infectivity, and changes in these residues reflect substitutions that are no longer targets of AgCNP reactivity. As a caveat to this interpretation, changes in D91 and F177 could alter the reactivity of AgCNP at a more distal site, as seen in the case of viruses that have lost sensitivity to neutralizing antibodies due to changes in more distal capsid locations outside of the actual antibody binding site [48]. The role of the individual AA changes in escaping AgCNP inactivation is not known, but it is noteworthy that both AAs were changed to more hydrophobic residues—D91V and F177L. It can be speculated that PHE-177 may be a key residue that is sensitive to AgCNP reactivity due to its planar aromatic ring, its position on the outside of the five-fold axis, and its known key interactions with other residues within the secondary structures in VP1. The other altered AA D91V may play a compensating role without any direct influence by AgCNP since it maps to a more flexible external loop outside of the core folds of VP1 (see Figure 4). Remarkably, Sherry et al., 1986 [40] have shown that the same D91V substitution is on the outside of RV14 virions, and this change occurs with RV14 variants which have gained resistance to neutralization by monoclonal antibodies—highlighting the shared mechanisms of inactivation and resistance between AgCNP and immune pathways.

What mechanism would drive surface changes in RV14 VP1 residues to become resistant to AgCNP? The RV14 capsid is a protein shell consisting of 12 pentamers—each pentamer composed of five copies of VP1–4 which form a central five-fold axis of symmetry surrounded by a deep canyon, which harbors many important AA residues involved in interactions with the cellular receptor ICAM-1 [36]. The location of AA changes in AgCNP-resistant RV14 at the surface of VP1 suggests that receptor–capsid interactions per se may not be directly disrupted by AgCNP, since the binding sites for many key receptor interactions are deep within the canyon and apparently do not change to gain AgCNP resistance. Instead, AgCNP may bind to or damage the easily accessible residue PHE-177, resulting in overall damaged virions. The AgCNP-resistant RV14 also had three additional nucleic acid changes in the genome that did not alter AA coding, and this may reflect compensating RNA structural changes to accommodate the AA changes in the resistant capsid. We have also found that sequential passage of the AgCNP-resistant virus did not give a reversion back to AgCNP-sensitivity. This is consistent with the extreme plasticity of RV14 for changes in capsid surface residues, as evidenced by the large number of distinct serotypes (>150) in circulation which can readily be selected to escape neutralization [49].

Table 1 lists some key structural properties of the viruses tested here for sensitivity to AgCNP inactivation, thus highlighting broad-spectrum anti-viral activity. Both RV14 and FCV are icosahedral non-enveloped RNA viruses of approximately the same size, but while FCV was easily inactivated by both AgCNP1 and 2, RV14 sensitivity was largely restricted to AgCNP2. This difference could reflect the relative high plasticity of RV14 capsids for tolerating changes to the virion surface [36,49] compared to FCV, or differences in the sensitivity of receptor–capsid interactions to disruption. It is generally considered that enveloped viruses are more sensitive to inactivation by antiviral agents as compared to non-enveloped viruses [2], due to exposure of the fragile lipid membrane to harsh external environmental conditions. However, this generality was not seen with the three enveloped viruses in our AgCNP studies—the related coronaviruses OC43 and SARS-CoV-2 had very different apparent AgCNP sensitivities, with OC43 being inactivated by both AgCNP1 and 2, whereas SARS-CoV-2 was only partially inactivated by AgCNP2. Similarly, PIV5 was only partially inactivated by exposure to AgCNP. Possible complicating factors in comparing relative sensitivities for these closely related viruses (e.g., OC43 versus SARS-CoV-2) could include relative differences in the following: (1) spike glycoprotein density (i.e., number of virus attachment proteins per virion), (2) strength of different receptor–spike interactions, and (3) composition of the lipid membrane for each virus. Given that each of these factors can be a reflection of the host cell the virus is derived from (e.g., during the budding process), an important future direction on the mechanism of AgCNP nanoparticles will be a systematic analysis of the role of the host cell in dictating relative sensitivity to these antiviral agents.

Understanding this dynamic relationship between virion composition and sensitivity to AgCNP-mediated inactivation could establish the foundation for the design of nanoparticles with higher specificity and potency. Our finding that AgCNP2 inactivation drove changes in capsid residues located away from the receptor binding region raises the hypothesis that nanoparticle anti-viral activity could be improved by engineering AgCNP particles to display viral receptors, such as ICAM1, LDLR, ACE2, and sialic acid residues. This strategy has been utilized previously with nanoparticles conjugated to sialic acid which inhibited influenza virus from binding to the receptor [56]. Coupling virus–receptor interactions and the potent anti-viral activity of silver-modified nanoceria is an attractive approach to improve upon the impressive and durable nanoparticle-based disinfection shown here.

An ideal property of hard surface disinfectants would be the ability to inactivate multiple pathogens with only one application. Here, we demonstrate proof-of-principle that a single dried application of either AgCNP1 or 2 to slides was sufficient to inactivate four sequential rounds of RV14 and FCV challenge, respectively. Importantly, AgCNP2-coated slides showed broad-spectrum antiviral activity against a mixture of viruses, with no apparent preference for RV14 inactivation over FCV inactivation in a mixed inoculum. These properties raise the hypothesis that AgCNP nanoparticles may have a capacity for regeneration of activity, which could be due to their ability to be self-contained ROS generators and scavengers [26,27]. These impressive properties of AgCNP nanoparticles highlight the potential for commercial and clinical applications in disinfectant surface coatings, as well as provide the foundation for new variations with increased specificity and potency.

## 4. Materials and Methods

### 4.1. Cells and Viruses

Cultures of RD, VeroE6, CV-1, HeLa, and Crandell–Rees Feline Kidney (CRFK) cells were grown in Dulbecco-modified Eagle medium (DMEM, Gibco, Thermo Fisher Scientific, Waltham, MA, USA) supplemented with 10% heat-inactivated fetal calf serum (HI FBS, Gibco, Thermo Fisher Scientific). HCT-8 cells were grown in Roswell Park Memorial Institute medium (RPMI 1640, Gibco, Thermo Fisher Scientific) supplemented with 10% HI FBS.

Human Coronavirus OC43 (ATCC, Manassas, VA, USA, catalog number VR-1558) was grown in HCT-8 cells at 33 °C as described previously [57]. OC43 stocks were quantified using a standard 50% Tissue Culture Infectious Dose (TCID_50_) assay on confluent RD cells in 96-well plates. Briefly, solutions were serially diluted in DMEM containing 0.38% Bovine Serum Albumin (BSA) as carrier protein. Cells were washed with PBS and incubated with diluted virus solutions for one hour at 33 °C. Cells were then washed and replaced with DMEM containing 2% HI FBS. At 4 days (d) post-infection (DPI) at 33 °C, cells were washed with PBS and stained with a crystal violet solution. TCID_50_ units were calculated by the Spearman & Kärber algorithm as previously described [58].

The USA-WA1/2020 strain of severe acute respiratory syndrome coronavirus 2 (SARS-CoV-2) was kindly provided by Ashley Brown, University of Florida [59], and was originally obtained from BEI Resources (catalog number NR52281, and NR-52285 Source Centers for Disease Control and Prevention). SARS-CoV-2 was grown in VeroE6 cells at 37 °C under BSL3 protocols approved by the UCF Biosafety Committee. At 3 DPI, media was clarified by centrifugation, and aliquots were frozen for storage at −80 °C. SARS-CoV-2 titers were determined by plaque assay on confluent VeroE6 monolayers using 0.4% agarose and DMEM containing 2% HI FBS at 37 °C. After 3 d, cells were stained with 1% crystal violet.

Human Rhinovirus 14 (RV14, ATCC, catalog number VR-284) was grown in HeLa cells at 33 °C, and RV14 stocks were determined by a TCID_50_ assay using confluent HeLa cells as described previously [28]. Feline Calicivirus (FCV, ATCC, catalog number VR-782) was grown in CRFK cells (ATCC, catalog number CCL-94) at 37 °C. At 1 DPI, media and dislodged cells were harvested and processed by three rounds of quick freezing in liquid nitrogen followed by rapid thaw in a 37 °C water bath. After clarifying cell debris, media was quickly frozen and stored in the −80 °C freezer. FCV virus titers were determined by plaque assay on CRFK cells.

Parainfluenza Virus Type 5 (PIV5) encoding the bacterial flagellin gene or Green Fluorescent Protein (GFP) inserted between the HN and L genes was grown in Vero cells [60] and tittered on CV-1 cells. Viruses were concentrated by centrifugation through a glycerol cushion; PIV5 (5 h (h); 25,000 rpm; SW28 rotor), OC43 (4 h, 24,000 rpm; SW28 rotor), and RV14 (2 h, 36,000 rpm; SW41 Ti rotor).

### 4.2. Nanoparticle Preparation and Virus Inactivation Studies

#### 4.2.1. Materials

Cerium nitrate hexahydrate (Ce(NO_3_)_3.6_H_2_O; 99.999% purity), silver nitrate (AgNO_3_, 99% purity), sodium hydroxide pellets (ACS reagent grade), and 3% hydrogen peroxide were procured from Sigma Aldrich, St. Louis, MO, USA. The 28–30% ammonium hydroxide was purchased from Alfa Aesar, Haverhill, MA, USA.

#### 4.2.2. AgCNPs Syntheses

AgCNP1: An amount of 1.74 g of cerium nitrate hexahydrate was dissolved in 40 mL of de-ionized water (>20 M Ω). An amount of 170 mg of silver nitrate was dissolved separately in de-ionized water [28]. The two aqueous solutions were combined and mixed via magnetic stirring. From here, stirring speed was increased to 450 rpm and the solution was titrated, drop-wise, with 0.4 M sodium hydroxide. After stirring for 20–30 min, the solution was washed 3× via centrifugation and re-suspension in pure de-ionized water. Then, the collected solution was titrated with ammonium hydroxide (final concentration: ~3 *v*/*v*%) and stirred for a day. Washing was repeated with the same parameters as performed earlier in the synthesis. The washed product was re-suspended in de-ionized water, ultra-sonicated, and allowed to stand overnight. The next day, well-dispersed particles were separated from any sedimented material.

AgCNP2: All chemicals were used without further purification for nanoparticle synthesis. Initially, 0.109 g (final concentration: 5 mM) of cerium nitrate hexahydrate was dissolved in de-ionized water (>20 MΩ) as a cerium source [28]. Parallelly, a 200 mM solution of aqueous silver nitrate was produced and added to the aqueous cerium nitrate solution to a final concentration of 1 mM. From here, the solution was continuously vortexed for several minutes to ensure complete dissolution of metal salts and to mix the metal ions uniformly. After the vortexing, 2 mL of 3% hydrogen peroxide was added into the cerium-silver co-solution, followed by vortexing. Finally, the oxidation reaction-initiated cerium-silver solution was stored in dark conditions at room temperature. After 3 weeks of aging, the solution was placed in a cellulose dialysis membrane and dialyzed against de-ionized water. Dialysate was exchanged every 12 h. This solution color changed from yellow to clear, indicating the reaction was completed and final-product nanoparticles had formed. The particles were maintained in glass bottles and stored in dark conditions at room temperature until use.

Nanoparticles were prepared, and studies in liquid were performed as previously reported [28]. Briefly, nanoparticles were ultrasonicated, diluted in inactivation buffer to the desired concentration as indicated in the Figure legends, and combined with the specified amount of viruses. Water controls were performed as vehicle controls. After indicated time incubations, remaining virus infectivity was quantified as described above. At the nanoparticle concentrations tested here, we observed no cell toxicity on the panel of indicator cells used.

Uncoated or AgCNP-coated glass slides were prepared by applying a specified concentration of nanoparticles as indicated in the Figure legends and allowing the solution to dry prior to virus challenge. Levels of infection of each respective virus described in the Figure legends were delivered to slides in triplicate in a biosafety hood. Titers of virus recovered as initial input infectivity were determined as time zero. At the incubation times indicated in the Figure legends, slides were vortexed and washed in an assay tube containing 0.5 mL DMEM/2% HI FBS. Samples were then analyzed for infectivity according to respective virus quantification assays as described above.

### 4.3. Hemagglutination Assays

Liquid nanoparticle reactions with pelleted PIV5 or crude OC43 were prepared as described in the Figure legends. After the indicated incubation time, reactions were serially diluted with PBS in round 96-well plates. Hemagglutination assays were performed as previously described [29] using washed chicken red blood cells (Lampire, catalog #7241409, Pipersville, PA, USA).

### 4.4. Sucrose Gradient Centrifugation

Liquid nanoparticle reactions with pelleted PIV5 and OC43 were assembled as described in the Figure legends. After the indicated incubation time, the reactions were layered on top of pre-formed 20–60% *w*/*v* sucrose (Thermo Fisher Scientific, catalog #177142500) gradients in NTE buffer as previously described [34]. After centrifugation (47,000 rpm, 45 min, 4 °C, Beckman Coulter SW55 Ti catalog #342194), 500 μL fractions were collected from the bottom of the gradient and lysed, while the remaining pellet was lysed directly in protein lysis buffer (recipe from Cell Signaling Technology, Danvers, MA, USA). The schematic of sucrose gradient outcomes in Figure 2A was created with BioRender.com (accessed on 20 June 2023).

### 4.5. Western Blotting

Protein lysates were analyzed by sodium dodecyl sulfate–polyacrylamide gel electrophoresis (SDS-PAGE) and western blotting as described previously [61]. PIV5 and OC43 sucrose gradient fractions were probed with rabbit antisera to the PIV5 P protein [62], or with mouse antibodies to coronavirus NP (1:2000, EMD Millipore, catalog #MAB9013, Burlington, MA, USA), respectively. Rhinovirus slide recovery media wash lysates were probed with mouse anti-VP3 (1:5000, The Native Antigen, catalog #4967, Kidlington, England). Blots were visualized by enhanced chemiluminescence (Thermo Fisher Scientific™ SuperSignal™ West Pico PLUS Chemiluminescent Substrate, catalog #PI34580) and visualization (Biorad, ChemiDoc MP, catalog #12003154 or film developer).

### 4.6. AgCNP2 Resistant Rhinovirus Selection and RNA Genome Sequencing

Approximately 5 × 10^5^ TCID_50_ units of parental RV14 were incubated with 0.1 mg/mL AgCNP2 for 1 h. Reactions were then diluted in DMEM containing 0.38% BSA and used to generate a virus stock in HeLa cells. These cycles of treatment and expansion were repeated for a total of 3 selection rounds with a nanoparticle concentration of 0.1 mg/mL. AgCNP2 concentration was then adjusted to 0.2, 0.1, and 0.15 mg/mL for the 4th, 5th, and 6th selection rounds, respectively. After the 6th selection round, RV14 was harvested and pelleted as described above. RNA extraction was performed using the Qiagen RNeasy Plus Mini kit (Qiagen, catalog #74134) according to manufacturer’s specifications.

After RNA quality control, 2 µg of RNA was shipped to CD Genomics where RNA integrity was analyzed using the Qubit RNA HS assay (Thermo Fisher Scientific), TapeStation (Agilent Technologies, Santa Clara, CA, USA), and Bioanalyzer 2100 Eukaryote Total RNA Nano (Agilent Technologies). RNA (1.5 µg) was utilized to prepare ssRNA libraries and sequencing was performed on the Illumina NovaSeq 6000 System with a library size of 300–500 bp, PE 250, and 100X, and paired-end reads were generated according to CD Genomics specifications. Files are available via NCBI BioProject accession PRJNA984283: Rhinovirus B raw sequence reads. Clean reads were then aligned to the RV14 reference genome NCBI Reference Sequence: NC_001490.1 [35]. Integrative Genomics Viewer (IGV) was utilized to identify mutations comparing parental and AgCNP2-resistant RV14 genomes [63]. Nucleotide sequences were then translated into amino acid sequences using ExPASy [64]. Protein modeling was performed using Phyre2 [37] and visualized on the PyMOL Molecular Graphics System [38].

### 4.7. Statistical Analyses

Unless otherwise indicated, values are the mean of three independent samples, with error bars representing the standard deviation. Statistical analysis was performed using GraphPad unpaired student’s *t*-test comparing untreated to the nanoparticle-treated samples. In all figures, * indicates *p*-value < 0.05, ** indicates *p*-value < 0.01, and *** indicates *p*-value < 0.001.

## Figures and Tables

**Table 1:** Summary of Virus Characteristics and Sensitivity to Dried Silver-Modified Nanoparticle- Mediated Inactivation.

Structure	Virus	Receptor ^a^	Particle Size Diameter (nm) ^b^	Dried AgCNP1 Sensitivity	Dried AgCNP2 Sensitivity
Non-enveloped + ssRNA	RV14	ICAM1 LDLR	15-30	-	+++
Non-enveloped + ssRNA	FCV	JAM-A	27-40	+++	+++
Enveloped + ssRNA	OC43	Sialic acid and others	120–160	++	+++
Enveloped + ssRNA	SARS-CoV-2	ACE2	100	-	+

^a^ Receptor references [10,30,32,50,51]. ^b^ Virion size references [32,52,53,54,55]. The range of virus sensitivity toward nanoparticle mediated inactivation is indicated by -, +, ++, and +++, where - is relatively insensitive and +++ is highly sensitive.

## Data Availability

Not applicable.

## Sample Availability

Samples of the compounds are not available from the authors.
